# Hepatitis B Virus Immunity Gap: A Six-Year Laboratory Data Review of Hepatitis B Serological Profiles in Gauteng Province, South Africa

**DOI:** 10.1155/2023/6374874

**Published:** 2023-05-17

**Authors:** Nonhlanhla Mbenenge, Kathleen Subramoney, Clement Gascua Adu-Gyamfi, Florette K. Treurnicht

**Affiliations:** ^1^School of Pathology, Faculty of Health Sciences, University of the Witwatersrand, Johannesburg, South Africa; ^2^Department of Virology, National Health Laboratory Service, Charlotte Maxeke Johannesburg Academic Hospital, Johannesburg, South Africa; ^3^Brain Function Research Group (BFRG), School of Physiology, Faculty of Health Sciences, University of the Witwatersrand, Johannesburg, South Africa; ^4^Center for Vaccines and Immunology, National Institute for Communicable Diseases (NICD), National Health Laboratory Service (NHLS), Johannesburg, South Africa

## Abstract

**Background:**

In 1995, the hepatitis B vaccine in South Africa was incorporated into the childhood expanded programme of immunization. We report on immunity gaps of laboratory-based hepatitis B virus (HBV) among patients in public facilities in Gauteng Province from 1st January 2014 to 31st December 2019. *Methodology*. We analyzed HBV serological data extracted from the National Health Laboratory Services Central Data Warehouse (NHLS CDW). A descriptive analysis was performed for hepatitis B surface antigen (HBsAg), antibodies to HBV core (anti-HBc) total, anti-HBc IgM, and antibodies to HBV surface antigen (anti-HBs) according to annual distribution, age groups, and sex.

**Results:**

The HBsAg positivity rate was 7.0% (75,596/1,095,561; *p*=0.001): 7.4% (96,532/944,077) in the 25 years and over age group and 4.0% (358/9,268 and 325/10,864) in the under 5 and 13–24 year age groups. The positivity rates of the other HBV serological markers were as follows: anti-HBc total was 37.0% (34,377/93,711; *p*  <  0.001), anti-HBc IgM was 2.4% (5,661/239,237; *p*=0.05), and anti-HBs was 37.0% (76,302/206,138; *p* ≤ 0.001). Naturally acquired HBV immunity was detected in 25.7% (11,188/43,536) of patients in the 25 years and over age group, and 9.7% and 8.2% (113/1,158 and 541/6,522) among those under 5 years and 13–24 year age group, respectively (*p*  <  0.001). Vaccine-induced immunity was 56.6% (656/1,158) in children under 5 years and 10.2% (4,425/43,536) among those 25 years and above (*p*  <  0.001). Fifty-six percent (29,404/52,581) of patients were HBV seronegative; predominantly among patients in the 13–24 year age group (60.6%; (3,952/6,522)) and 25 years and over (56.3% (24,524/43,536)) (*p*=<0.001).

**Conclusion:**

The HBV infection seroprevalence remains high in South Africa, with Gauteng province having high intermediate endemicity. However, the HBV immunity gap has shifted from younger children to older children and adults.

## 1. Introduction

Hepatitis B virus (HBV) infection is a potentially life-threatening, vaccine-preventable infection. It may acutely present as *icteric hepatitis* and less commonly as *fulminant hepatitis* or as chronic *hepatitis* that results in liver cirrhosis, liver failure, and hepatocellular carcinoma [1, 2]. In 2019, an estimated 316 million people globally became chronically infected with HBV [3]. According to the World Health Organization (WHO) global hepatitis report, the African region was estimated to have 60 million chronic HBV-infected people, and tSe sub-Saharan region carried the highest disease burden. Furthermore, HBV infection was estimated to account for 88,000 deaths annually in Sub-Saharan Africa [1]. A systematic review of HBV prevalence by Schweitzer et al. estimated HBV prevalence in South Africa was high at 6.7%, and the number of people living with chronic HBV infection accounted for about 3.5 million [4].

Hepatitis B infection is acquired from HBV-infected bodily fluid (such as blood, saliva, or breastmilk) of an infected person [2]. The modes of HBV transmission may follow various routes, which impact the geographical prevalence [2, 5]. The perinatal route (mother-to-child transmission) is the predominant mode of HBV spread globally [5]. In intermediate (2–8%) and high (>8%) HBV-endemic countries, such as South Africa, the predominant mode of HBV transmission is the perinatal and horizontal route [2, 5, 6]. Furthermore, the majority of HBV infections occur in early childhood during the first five years of life [5–7].

Chronic hepatitis B infection has been defined as the persistence of the hepatitis B surface antigen (HBsAg) in the blood for six months or more [2]. Broad evidence available indicates that the risk of developing chronic hepatitis B infection is age-dependent [1–4]. Without effective HBV prophylaxis, about 90% of children who acquire HBV infection neonatally will become chronic carriers of HBV. About 20–60% amongst those are in the under 5 years of age group. HBV chronicity is estimated at 5–10% of older children (>5 years of age) and less than 5% of those infected after the age of 20 [1, 6, 7].

An effective hepatitis B vaccine has been available since the early 1980s. In addition, research has shown a persistent protective immune response post-vaccination for at least 20 years, with evidence of protective immunity in more than 90% of people after three doses [6, 8]. In South Africa, the hepatitis B vaccine has been part of the childhood expanded programme of immunization (EPI) since 1995 [8, 9]. At its inception, the hepatitis B vaccine was a monovalent antigen administered to infants at 6, 10, and 14 weeks of life [8–10]. However, in 2015, it was replaced by a more “baby-friendly” hexavalent vaccine administered at 6, 10, and 14 weeks, and the fourth dose at 18 months of life [8]. Nevertheless, HBV vaccination coverage has been variable. In 2019-2020, 83.5% of infants were reported to have received the third dose of the hepatitis B vaccine, while the full vaccine coverage declined to 76.8% by the fourth dose [11, 12]. Meanwhile, the hepatitis B vaccine coverage among healthcare workers in Gauteng was reported to range from 15.4% to 90% and over [11].

The positive impact of the hepatitis B vaccination programme has been the elimination of chronic hepatitis B, with a greater than 60% decline in HBV prevalence [8, 10, 11]. However, the prevalence of HBV remains high, depending on locality and population group [13]. Some studies estimated the prevalence of HBV monoinfection in South Africa at 1.0% in urban areas and approximately 10% in rural areas [14]. A population-based household survey study conducted in 2014-2015 reported an HBsAg prevalence of 4.0% in the 15–49 year age group in the KwaZulu-Natal province of South Africa [14], while the national HBV prevalence rate study showed the highest prevalence of HBV in Gauteng province [15]. Furthermore, a study by Prabdial-Sing et al. on children under 15 years of age in South Africa showed that 2.9% of the children had exposure to HBV [16].

Recently, the World Health Organisation (WHO) proposed strategies to eliminate HBV by 2030 [17]. The proposed strategies aim to reduce the incidence of chronic hepatitis B by curbing the disease in children under five years by 90.0% and HBV mortality by 65.0% [18]. Therefore, there is a need to identify existing gaps that negatively impact achieving HBV prevention, control, and elimination in South Africa.

Thus, this study aims to describe the epidemiological challenges driving the HBV prevalence (immunity gaps) determined from laboratory-based diagnosis of hepatitis B viral HBV infection among patients attending public health care facilities in Gauteng Province, South Africa, from January 1, 2014, to December 31, 2019.

## 2. Materials and Methods

### 2.1. Study Design and Site

This was a retrospective descriptive cross-sectional study on qualitative hepatitis B serological diagnosis among patients who sought care at public health facilities in Gauteng Province from 1 January 2014 to December 31 December 2019. This study was conducted in the Department of Virology of the National Health Laboratory Service (NHLS) at Charlotte Maxeke Johannesburg Academic Hospital and the University of the Witwatersrand, Johannesburg, Gauteng Province, South Africa.

### 2.2. Study Population and Sample Selection

The study included the laboratory results of patients of all ages with HBV serological testing for the HBsAg, antibodies to HBV core (anti-HBc) total, anti-HBc IgM, and antibodies to HBV surface antigen (anti-HBs) performed at any of the NHLS Pathology laboratories in the Gauteng Province. Anonymized study data were obtained from the NHLS Central Data Warehouse (CDW). The inclusion criteria in the study were patient results without missing or incomplete data for age and sex and a validated positive, negative, or equivocal result recorded. Equivocal results were interpreted as neither negative nor positive. The HBV serological datasets without date of birth (age) and a missing observation for sex were excluded, including data generated from healthcare facilities outside the Gauteng province. The patient records were retrieved per year from 2014 to 2019. Each patient's annual testing episodes were identified using a unique patient folder number, which was then used to deduplicate the annual datasets.

### 2.3. Laboratory Testing and Interpretation

Serological testing for HBsAg, anti-HBc total, anti-HBc IgM, and anti-HBs was conducted using the enzyme-linked immunosorbent assay on automated instruments: Architect (Abbott, Germany) or COBAS (Roche, USA) according to the manufacturer's instructions. Sample results that were equal or greater than sample cutoff (S/CO), as specified in the manufacturer's instruction or the laboratory standard operating procedure, were considered to be positive.

The S/CO ≥ 1 for HBsAg, anti-HBc total, and anti-HBc IgM was considered positive. In addition, the anti-HBs titre greater or equal to 10 milli-international units per mil (mUI/ml) were assigned as positive and were associated with a measurable, significant correlate of protection [19].

The descriptive interpretation of the test results was as follows: (i) anti-HBc IgM positive results in the presence of HBsAg and anti-HBc positive results are indicative of acute HBV infection; (ii) HBsAg positive, anti-HBc total positive, and anti-HBs negative results are indicative of HBV infection; (iii) HBsAg negative, anti-HBc total positive, and anti-HBs positive results are indicative of naturally acquired HBV immunity; (iv) HBsAg negative, anti-HBc total negative, and anti-HBs positive results were indicative of HBV vaccine-induced immunity; and (v) HBV seronegativity was indicated by HBsAg negative, anti-HBc total negative, and anti-HBs negative test results [19].

### 2.4. Statistical Analysis

Variables included were the “test date,” “year,” “test site or location,” “date of birth,” “age,” “sex,” “HBV serology test-set markers,” (HBsAg, anti-HBc total, anti-HBc IgM, and anti-HBs) and “test results.” For descriptive analysis, we used Microsoft Excel 2016 and GraphPad Prism (version 7.0, California, USA). The parametric data were presented as the mean and nonparametric data as the confidence interval and the interquartile range. The *p* value was generated using the *t*-test (paired two-sample for the means). The estimated positivity rate for each test type was based on the number of positive cases among the total population.

## 3. Results

### 3.1. Demographic Characteristics of the Study Population

From 2014 to 2019, a total of 1,403,777 HBV serology datasets were registered on the NHLS CDW for patients who sought care at public health facilities in Gauteng. Of these, a total of 1,326,614 patients' HBV serology datasets with a registered date of birth (age) and sex were included in the study population. The study population was divided into four age groups (Table 1).

The total number of patients tested has fluctuated over the years. However, there was an increase from 2014 (5.0%; 66,974/1,326,614) to 2019 (22.1%; 292,900/1,326,614) among all age groups (Table 1). The 25 years and above age group represented 86.0% (1,141,567/1,326,614) of the study population, whereas the 13–24 age group represented 11.8% (157,152/1,326,614). While those of the 5–12 age group as well as those under-5 age group each accounted for 1.0% ((14,547/1,326,614) and (13,348/1,326,614)) (Table 1). The highest testing rates (60.5%; 803,076/1,326,614) were from females, and 39.5% (523,538/1,326,614) were from males (Table 1).

### 3.2. Testing Rate of HBV by Serological Markers

#### 3.2.1. Hepatitis B Virus Surface Antigen

Of the 1,326,614 patient results, HBsAg testing accounted for 1,095,561 (82.6%). The total number of HBsAg testing performed increased annually from 4.6% (49,963/1,095,561) in 2014 to 23.3% (255,737/1,095,561) in 2019 (Supplementary Table 1). The majority (86.2% (944 077/1,095,561)) of the patients tested for HBsAg were of the age group 25 years and over. In contrast, among patients aged 0–24 years, the HBsAg testing rate was 12.0% (131,352/1,095,561) among the 13–24 age group and 1.0% (9,268/1,095,561 and 10,864/1,095,561) in the under 5 and 5–12 age groups, respectively.

The overall HBsAg positivity rate was 7.0% (75,596/1,095,561) (Table 2). However, the annual HBsAg positivity rate decreased from 8.0% (4,006/49,963) in 2014 to 6.1% (15,495/255,737) in 2019. The positive HBsAg tests were high at 7.4% (69,532/944,077) among patients in the 25 and over age group. In comparison, 4.0% was observed in patients aged 0–24 years. Of these, 4.0% positives were observed for both patient age groups, the under 5 and 13–24 years (358/9,268 and 325/10,864), and 3.2% (5,381/131,352) for the age group 5–12 years. While females were in the majority, the HBsAg positivity rate was significantly higher at 9.8% (41,795/425,521; IQR 16 (50−34)) among males compared to 5.0% (33,801/670,040; IQR 18 (48−30)) (*p*=0.001) in females (Table 2).

#### 3.2.2. Anti-HBc Total Antibodies

Of the 1,326,614 patient results, anti-HBc total was reported for 93,711 (7.0%) patients. From 2014 to 2016, the total number of patients tested for anti-HBc total increased from 15.6% (14,606/93,711) to 24.5% (22,955/93,711), but declined to 13.8% (12,916/93,711) in 2017 and successive years to 12.4% (11,591/93,711) in 2019 (Supplementary Table 1). Patients aged 25 years and over accounted for 83.8% (78,598/93,711) of anti-HBc Total testing, followed by those in the 13–24 age group at 11.7% (10,957/93,711) and 2.2% (2,092/93,711 and 2,063/93,711), respectively, among those aged less than 5 years and 5–12 age group.

The overall anti-HBc Total positivity rate was 37.0% (34,377/93,711) (Table 3). The annual anti-HBc total positivity rate increased each year from 33.7% (4,923/14,606) in 2014 to 40.4% (4,763/11,791) in 2018. However, it declined to 37.0% (4,288/11,591) in 2019. The anti-HBc total positivity rate was higher, at 40.8% (32,104/78,599) in the 25 years and over age group. In patients aged 0–24 years, the anti-HBc Total positivity rate was 16.3% (1,786/10,957) for the 13–24 age group and 15.0% and 8.3% for the under 5 and 5–12 year age groups (313/2,092 and 171/2,063), respectively. Although females dominated, the anti-HBc total positivity rate was higher in males ((43.0%; 17,351/40,412); IQR 16 (50−34)) compared to females ((32.0%; 17,026/53,299); IQR 18 (48−30)) (*p*=0.006).

#### 3.2.3. Anti-HBc IgM Antibodies

Of the 1,326,614 patient results, the anti-HBc IgM was reported for 239,237 (18.0%) patients. The annual anti-HBc IgM increased from 7.0% (16,491/239,237) in 2014 and reached a peak of 23.7% (56,804/239,237) in 2017. In the successive year, 2018 and 2019, it declined (17.7%, 42,316/239,237 and 13.5%, 32,204/239,237) (Supplementary Table 1). The majority, 87.0% (208,095/239,237), of anti-HBc IgM positive results were from patients in the age group 25 years and over, and those in the 13–24 year age group at 10.5% (25,200/239,237). The lowest presentation was from patients under 5 years of age and those in the 5–12 year age group at 1.3% and 1.2% (3,154/239,237 and 2,788/239,237), respectively.

The overall anti-HBc IgM positivity rate was 2.4% (5,661/239,237) (see Table 4 below). However, there was a downward trend in the annual anti-HBc IgM positivity rate from 4.4% (718/16,491) in 2014 to 2.5% (1,293/52,195) in 2016 and was stagnant at 2.0% (*p*=0.05) in the successive years (2017–2019). The anti-HBc IgM positivity rate was high in the 13–24 year age group at 3.4% (865/25,200) and those in the 25 years and over age group at 2.3% (4,750/208,095). Moreover, it was 0.7% (23/3,154) and 0.8% (23/2,788) for the under 5 and 5–12 year age groups, respectively. There was no difference in the anti-HBc IgM positivity rate among females ((2.0%; 2,719/137,061); IQR 14 (39−29)) and males ((3.0%; 2,942/102,176); IQR 14 (41−27)) (*p*=0.364).

#### 3.2.4. Anti-HBs Antibodies

Of the 1,326,614 patient results, the anti-HBs were tested in 206,138 (15.5%) patients. The annual percentage of patients tested for anti-HBs increased from 10.0% (20,237/206,138) in 2014 to 20.1% (41,392/206,138) in 2016, followed by a plateau that remained at around 18.0% in successive years (2017: 37,970/206,138; 2018: 38,165/206,138; and 2019: 36,575/206,138) (Supplementary Table 1). A significant proportion (83.5%; 172,061/206,138) of patients tested was in the 25 year and over age group. Among patients aged 0–24 years, 12.2% (25,229/206,138) were in the 13–24 year age group, and 2.0% were in the under 5 and 5–12 year age groups (4,406/206,138 and 4,442/206,138), respectively.

The overall anti-HBs positivity rate was 37.0% (76,302/206,138) (Table 5). The annual anti-HBs positivity rate showed a downward trend from 37.0% (7,480/20,237) in 2014 to 35.4% (14,636/41,392) in 2016. However, it peaked in 2017 (37.6%, 14,284/37,970) and gradually increased to 38.7% (14,140/36,575) by 2019. There was no difference in the anti-HBs positivity rate among the age groups (under 5 year age group: 62.0%, 2,722/4,406; 5–12 year age group: 54.0%, 2,397/4,442; 13–24 year age group: 37.7%, 9,506/25,229; and 25 year and above age group: 36.0%; 61,677/172,061) (*p*=3.38) (Table 5). Females had a higher anti-HBs positivity rate at 38.3% (46,112/120,319; IQR 20 (46−26)) compared to males at 35.2% (30,190/85,819; IQR 20 (49−29)) (*p*=0.002).

### 3.3. Descriptive Clinical HBV Serology Results

Of the 1,326,614 patient results, the HBsAg, anti-HBc total, and anti-HBs serology markers combined were available for 56,294 (4.2%) patients. The distribution by age group, sex, and tests by year of these patients (HBV-infected, naturally acquired and vaccine-induced immunity, and seronegative HBV) is illustrated in Supplementary Table 2. After excluding patients with equivocal results, the included study population in the descriptive clinical HBV positivity rate analysis was 52,581 (4.0%) (Table 6).

**Figure 1:**
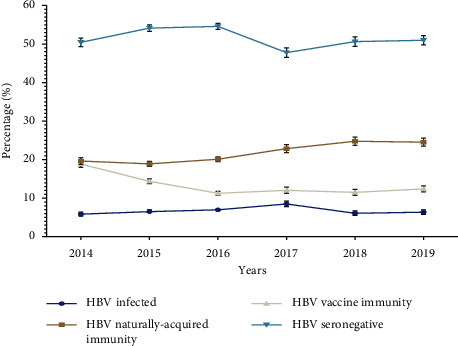
The proportion of patients with either HBV infection, naturally acquired immunity, vaccine-induced immunity, or seronegative by year in Gauteng Province, South Africa, from 2014 to 2019.

**Figure 2:**
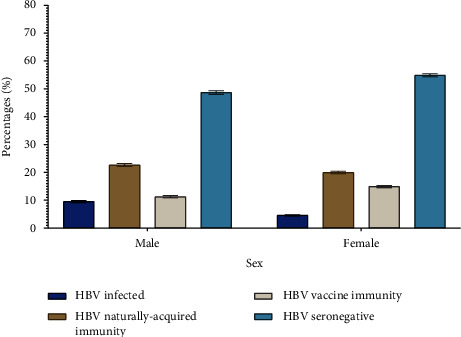
The proportion of patients with either HBV infection, naturally acquired immunity, vaccine-induced immunity, or seronegative by sex in Gauteng Province, South Africa, from 2014 to 2019.

Overall, 7.0% (3,766/52,581) of patients had positive HBsAg, positive anti-HBc total, and negative anti-HBs test results (HBV-infected) (Table 6). The number of HBV-infected patients increased from 6.0% (481/8,216) in 2014 to 8.50% (525/6,178) in 2017, followed by a decline in subsequent years to 6.4% (429/6,736) in 2019 (*p*  <  0.001) (Figure 1). The HBV positivity rate was significantly higher at 8.0% (3,431/43,536) in the 25 years and over age group and 4.2% (272/6,522) in the 13–24 year age group compared to patients in the under 5 and 5–12 year age groups (2.0% (21/1,158) and 3.1% (42/1,333) (*p*  <  0.001), respectively) (Table 6). The HBV infection rate was significantly higher among males (9.5%, 2,280/23,995) compared to females (4.6%; 1,486/32,299) (*p*  <  0.001) (Figure 2).

Naturally acquired HBV immunity (negative HBsAg, positive anti-HBc total, and positive anti-HBs) were identified among 22.6% (11,887/52,581) of the patients (Table 6). The number of patients with naturally acquired HBV immunity increased from 19.6% (1,613/8,216) in 2014 to 24.8% (1,607/6,491) in 2018 and remained stable in 2019 (24.5%; 1,653/6,736) (*p*  <  0.001) (Figure 1). The positivity rate of naturally acquired HBV immunity was 26.0% (11 188/43,536) in the 25 years and over age group. While among patients aged 0–24 years, the naturally acquired HBV immunity was high at 10.0% (113/1,158) in the under 5 year age group and 8.3% (541/6,522) in the 13–24 year age group, and least (3.4%) in the age group 5–12 (*p*  <  0.001) (Table 6). Evidence of naturally acquired HBV immunity was seen in more males (22.7%; 5,435/23,995) compared to 20.0% (6,452/32,299) (*p*  <  0.001) than females (Figure 2).

Fourteen percent (7,524/52,581) of patients had negative HBsAg, negative anti-HBc total, and positive anti-HBs test results (hepatitis B vaccine-induced immunity) (Table 6). A decline was observed in the number of patients with evidence of hepatitis B vaccine immunity from 18.8% (1,548/8,216) in 2014 to 11.3% (1,718/15,242) in 2016. However, it remained stable at 12.0% for the successive years (2017–2019) (*p*  <  0.001) (Figure 1). The vaccine-induced immunity was highest, 56.7% (656/1,158), in the under 5-year age group. Furthermore, it was also significantly high among those in the 5–12 and 13–24 year age groups (51.5%; 686/1,333 and 27.0%; 1,757/6,522), respectively. However, the hepatitis B vaccine-induced immunity was low (10.0%; 4,425/43,537) for the 25 years and above age group (*p*  <  0.001) (Table 6). The positivity rate of hepatitis B vaccine-induced immunity was higher at 15.0% (4,820/32,299) in females compared to males (11.3%, 2,704/23,995) (*p*  <  0.001) (Figure 2).

The negative results for HBsAg, anti-HBc total, and anti-HBs (seronegative HBV) were identified among 56.0% (29,404/52,581) of patients (Table 6). The proportion of seronegative HBV patients increased from 50.4% (4,143/8,216) in 2014 to 54.6% (8,321/15,242) in 2016. A decline to 47.8% (2,951/6,178) followed in 2017; however, an upward trend was evident for 2018 and 2019 (50.6%, 3,261/6,491 and 51.0%, 3,434/6,736), respectively (*p*  <  0.001) (Figure 1). The majority of these patients were in the 13–24 year age group at 60.5% (3,952/6,522), followed by the 5–12 and under 5-year age groups at 42.0% and 31.0% (560/1,333 and 368/1,158) (*p*  <  0.001), respectively (Table 6). The HBV seronegativity was higher for females in contrast to males at 55.0% (17,727/32,299) and 48.7% (11,677/23,995) (*p*  <  0.001), respectively (Figure 2).

## 4. Discussion

This study is a retrospective analysis of the HBV serological data to identify immunity gaps since the start of hepatitis B vaccination in South Africa. We reviewed the reactivity of HBV serology markers among patients who attended urban public health facilities in Gauteng province. Testing for HBV infection may have been necessary for several reasons. In children, HBV infection predominantly presents as subclinical. Therefore, HBV testing for these may have been for a medical workup for a medical condition or screening for perinatal maternal infection [15]. In contrast, adults are invariably symptomatic, with only ≤5.0% developing chronic disease [1, 20]. Therefore, in a country with a high HIV prevalence, testing may have been part of HIV management since HIV-infected people have the highest rates of HBV infection [21]. The study population consisted predominantly of females, and their ages varied, with the 25-year-old and older age group comprising the majority. The predominance of females in this study may be associated with a higher probability of symptomatic infection and health-seeking behaviour compared to males [15].

Overall, our data showed an HBsAg seroprevalence of 7.0% among our study population. The detection of HBsAg in serum confirms the diagnosis of HBV infection [20–22]. A similar study on hepatitis B seroprevalence across South Africa reported a median HBV prevalence of 8.3% for the same province [15]. Although the annual total of positive cases of HBsAg showed a downward trend, the high seroprevalence observed in this study highlights that South Africa is still an intermediate-to-high HBV endemic country [20–22]. Countries with ≥8.0% HBV prevalence are regarded as having high endemicity, while those with a prevalence of less than 2.0% are of low endemicity [20, 23]. In 2014 and 2015, the overall prevalence of HBV was lower in KwaZulu-Natal Province at 4.0% among 15- to 49-year-olds [14]. Our finding showed an HBsAg seroprevalence almost double of what was expected for the Gauteng province since it is the country's business hub and people migrate from other provinces to Gauteng for work and better healthcare services.

Among the age groups eligible for hepatitis B vaccination (0–24 years), the HBV seroprevalence in the under-5 and 5–12-year age groups was 2.0% and 3.1%, respectively. These findings were in agreement with a similar study by Moonsamy et al. [15], where the researchers reported a low to intermediate endemicity prevalence between 2.0% and 5.0% in children under 15 years of age. The risk of developing chronic HBV infections is age-dependent. Perinatal and neonatal HBV infection results in up to 90% of chronic infection and decreases to 30.0%–50.0% in children between one and four years [6, 20, 23]. Early-life infection adds to the burden of chronic HBV infection despite the availability of effective vaccines. South Africa has not yet implemented the hepatitis B vaccine birth dose. Therefore, the cases of HBsAg in children under 5 years may stem from perinatal acquisition of HBV and horizontal transmission, which is the main route of HBV transmission in South Africa and the African region [8, 20].

This study found a high HBV seroprevalence in males compared to females. A similar finding for HBsAg and hepatitis B e-antigen (HBeAg), a marker for active HBV replication, was reported by Samsunder et al. in the HBV community seroprevalence survey conducted in KwaZulu-Natal in 2014-2015 [14]. Therefore, the higher seroprevalence of HBV among men reported here is not unexpected. This finding was consistent with reported reviews that males are a risk factor for chronic HBV infection and rapid progress to HBV-associated hepatocellular carcinoma [13, 24, 25]. The potential mechanisms for gender disparity in this study are not clear; nevertheless, a possible contributing factor may be an interplay between sex hormones and the immune response, as females have a higher proneness to symptomatic viral infection related to their intense inflammatory responses [24].

Since antibodies to the hepatitis B core antigen persist for life, their presence provides evidence of natural exposure to circulating HBV. Therefore, these antibodies are found in patients with chronic HBV infection and individuals who recovered from the infection (naturally-acquired hepatitis B immunity) [19, 26]. In this study, naturally-acquired hepatitis B immunity was observed in 23.0% of the patients (Table 6). Although the highest seroprevalence of those with naturally-acquired HBV immunity was among those 25 years of age and older, the 10.0% of naturally-acquired HBV immunity among children under 5 years of age was also significant. This analysis may attest to an existing gap in preventing perinatal transmission through maternal HBsAg screening and treatment. Additionally, providing post-exposure prophylaxis to newborns whose mothers are HBsAg positive [21, 22], these gaps could be reduced by implementing HBV point-of-care diagnostic testing, the *de facto proviso* for HBsAg diagnosis during pregnancy, and vaccination strategies, i.e., the introduction of hepatitis B birth dose as outlined in the current Viral Hepatitis Guidelines for South Africa [21].

The overall positivity rate of anti-HBc IgM was 2.4%. The anti-HBc IgM detected in patients with positive HBsAg and anti-HBc Total indicates acute or reactivation of latent HBV infection [19, 21, 26]. During the study period, the annual anti-HBc IgM positivity rate showed a downward trend and was less than 1.0% among those under 5 and in the 5–12 year age groups. This finding may demonstrate the positive impact of the hepatitis B vaccination programme. Nonetheless, a higher anti-HBc IgM positivity rate was observed in the 13–24 age group (3.4%) and those 25 years and over (2.3%). Therefore, the high anti-HBc IgM levels in the 13–24 age group and those 25 years and over may have been overestimated since reactivation of latent HBV infection may cause low-level anti-HBc IgM positivity. However, our findings may suggest engagement in high-risk behaviours such as unprotected sex, multiple sexual partners, or shared use of intravenous drug injections [15]. Since the two age groups mentioned above are also associated with high HIV prevalence (covariable) in South Africa, active management strategies to prevent secondary transmission of both HBV and HIV are needed [26–28].

The overall positivity rate of anti-HBs was 37.0%. The presence of anti-HBs in the absence of HBsAg and anti-HBc implies immunity against HBV infection through vaccination [8, 21, 29]. This study found that the overall seroprevalence of hepatitis B vaccine-induced immunity was 14.0%. This vaccine-induced immunity was higher at 57.0% and 51.5% in the under-5 and 5–12 year age groups, respectively, but lower than expected. Previous South African studies reported vaccine-induced immunity rates of 85.7% and 78.1% among HIV-negative and HIV-positive 5–24-month-old babies, respectively [10, 30]. Our findings were also lower than reported for a community-based surveillance South African study conducted in 2013 among children with febrile rash, which reported a vaccine-induced immunity of 81.8% for infants and 80.2% for children, respectively; however, their sample size was small [16]. The lower hepatitis B vaccine-induced seroprotection observed in our study may imply missed vaccination, biological nonresponse, or waning anti-HBs concentration, which tends to fall off quickly after the prime immunization course [31]. In addition, a review study on undetectable anti-HBs antibodies in HIV-1-infected individuals reported a lower HBV vaccine response and a rapid loss of protection among these patients, including HIV-exposed but uninfected children, compared to HIV-unexposed children [32]. Therefore, in a country with a high burden of HIV infection and HIV-exposed children, such as South Africa, serological monitoring of anti-HBs antibodies should be part of the HBV preventative strategies.

In this study, 56.0% of patients were seronegative for all HBV markers (HBsAg, anti-HBc total, and anti-HBs). These results may indicate no exposure to HBV infection or a lack of hepatitis B vaccine-induced immunity, perhaps due to a lack of vaccination, vaccine nonresponse, or waning immunity [29, 31, 33]. Of these, the age group 25 years and over accounted for 56.0%. However, the subgroup analysis indicated a higher percentage, 83.4%. The high seronegative rate among the 25-year-old and older age group was expected, as these patients were not eligible for EPI, which began in 1995. However, the HBV seronegative rate among patients in the 13–24 age group was also significantly higher. Similar findings were reported by Prabdial-Sing et al. [16] in children younger than 15 years. Of these, some seronegative patients may benefit from hepatitis B vaccination. However, booster doses are not recommended for those who have completed their vaccination doses, as this does not imply a loss of protection since an anamnestic response is induced following the booster dose [22, 31].

Our study was possibly biased because the design used seroprevalence estimates obtained from patients who sought medical care in public health facilities but were not randomly selected. Therefore, our laboratory data may not be representative of the general population. However, since public health facilities cater to 80% of the national population, 80.0% of HBV serological testing is primarily conducted by national health laboratories. Additionally, our findings were in agreement with other similar studies. Therefore, the fundamental strength of this study is its explanatory power in understanding the changing HBV epidemiology landscape in the South African context and the need to strengthen the prevention and control of HBV infection among different age groups.

## 5. Conclusion

The introduction of the hepatitis B vaccine in the South African EPI schedule has positively impacted the under 5-year HBV seroprevalence. Although there are gaps in the prevention of vertical and horizontal transmission, optimal implementation of the South African national guidelines for managing viral hepatitis, i.e., implementation of the HBV birth dose and optimum EPI coverage, may lead to the targeted goal of HBV prevalence <1.0% for children under 5 years. Therefore, cost-effective prevention and control strategies such as public awareness and education, HBV screening, and vaccination for high-risk age groups are recommended for immediate implementation. However, the HBV positivity rate of 7% in the Gauteng province still indicates high intermediate endemicity. From these data, we conclude that the HBV immunity gap has shifted from younger children to older children and adults.

## Figures and Tables

**Table 1:** Demographic characteristics of patients who sought care for HBV serological diagnosis in Gauteng Province, South Africa, from 2014 to 2019.

Variables	2014 *N* = 66,974	2015 *N* = 180,739	2016 *N* = 253,969	2017 *N* = 280,914	2018 *N* = 251,118	2019 *N* = 292,900	Total *N* = 1,326,614
	*n* (%)	*n* (%)	*n* (%)	*n* (%)	*n* (%)	*n* (%)	*n* (%)
*Age group (years)*							
<5	1,506 (2.3)	2,045 (1.1)	2,218 (0.9)	2,570 (1.0)	2,646 (1.1)	2,363 (0.8)	13,348 (1.0)
5–12	1,470 (2.2)	2,370 (1.3)	2,672 (1.1)	2,888 (1.0)	2,547 (1.0)	2,600 (0.9)	14,547 (1.1)
13–24	8,338 (12.5)	20,692 (11.4)	28,718 (11.3)	31,697 (11.0)	28,881 (11.5)	38,826 (13.3)	157,152 (11.8)
≥25	55,660 (83.0)	155,632 (86.1)	220,361 (87.0)	243,759 (86.8)	217,044 (86.4)	249,111 (85.0)	1,141,567 (86.1)

*Sex*							
Female	40,616 (60.6)	107,956 (60.0)	153,612 (60.5)	169,880 (60.5)	152,110 (60.6)	178,902 (61.1)	803,076 (60.5)
Male	26,358 (39.4)	72,783 (40.0)	100,357 (39.5)	111,034 (39.5)	99,008 (39.0)	113,998 (39.9)	523,538 (39.5)

**Table 2:** The distribution of positive HBsAg per year across all age groups and sex in Gauteng Province, 2014–2019.

Variable	Total number	HBsAg		
	*N*	*n*	% (95% CI)	*p* value
*Age group*				
<5	9,268	358	3.8 (7.6–12.6)	*p* = 0.001
5–12	10,864	325	3.2 (6.4–13.6)	
13–24	131,352	5,381	4.1 (57.6–65.4)	
≥25	944,077	69,532	7.4 (52.1–66.7)	

*Sex*				
Female	670,040	33,801	5.0 (67.2–78.1)	*p* = 0.001
Males	425,521	41,795	9.8 (52.1–79.3)	

*Year*				
2014	49,963	4,006	8.0 (53.7–63.8)	*p* = 0.001
2015	147,290	10,638	7.2 (61.0–69.4)	
2016	209,900	15,580	7.4 (65.7–73.8)	
2017	226,444	16,277	7.2 (78.3–92.2)	
2018	206,227	13,600	6.6 (55.5–66.9)	
2019	255,737	15,495	6.1 (58.1–70.1)	
Total	1,095,561	75,596	6.9	

**Table 3:** The distribution of positive anti-HBc total per year across all age groups and sex in Gauteng Province, 2014–2019.

Variable	Total number	Anti-HBc total		
	*N*	*n*	% (95% CI)	*p* value
*Age group*				
<5	2,092	313	14.9 (7.7–11.1)	*p* = 0.002
5–12	2,063	171	8.3 (2.5–4.4)	
13–24	10,957	1,786	16.3 (7.4–8.7)	
≥25	78,599	32,104	40.8 (33.4–44.2)	

*Sex*				
Female	53,299	17,023	32.0 (39.8–40.2)	*p* = 0.006
Males	40,412	17,351	43.0 (42.3–42.7)	

*Year*				
2014	14,606	4,923	33.7 (18.9–20.5)	*p* < 0.001
2015	19,852	7,036	35.4 (18.2–25.8)	
2016	22,955	8,335	36.3 (19.5–21.7)	
2017	12,916	5,029	39.0 (23.7–27.8)	
2018	11,791	4,763	40.4 (23.7–28.2)	
2019	11,591	4,288	37.0 (23.5–28.8)	
Total	93,711	34,374	37.0	

**Table 4:** The distribution of positive anti-HBc IgM per year across all age groups and sex in Gauteng Province, 2014–2019.

Variable	Total number	Anti-HBc IgM		
	*N*	*n*	% (95% CI)	*p* value
*Age group*				
<5	3,154	23	0.7 (2.1–4.3)	*p* = 0.002
5–12	2,788	23	0.8 (2.1–4.2)	
13–24	25,200	865	3.4 (26.2–27.2)	
≥25	208,095	4,750	2.3 (42.3–55.6)	

*Sex*				
Female	137,061	2,719	2.0 (66.1–81.3)	*p* = 0.364
Males	102,176	2,942	3.0 (69.1–87.5)	

*Year*				
2014	16,491	718	4.4 (18.0–19.7)	*p* = 0.05
2015	39,227	1,036	2.6 (13.8-14.9)	
2016	52,195	1,293	2.5 (10.7–11.7)	
2017	56,804	1,082	2.0 (11.2–12.8)	
2018	42,316	880	2.1 (10.7–12.3)	
2019	32,204	652	2.0 (11.6–13.2)	
Total	239,237	5,661	2.4	

**Table 5:** The annual distribution of anti-HBs positive cases per year across all age groups and sex in Gauteng Province, 2014–2019.

Variable	Total number	Anti-HBs		
	*N*	*n*	% (95% CI)	*p* value
*Age group*				
<5	4,406	2,722	62.0 (27.6–32.7)	*p* = 3.38
5–12	4,442	2,397	54.0 (38.5–44.6)	
13–24	25,229	9,506	37.7 (57.6–60.1)	
≥25	172,061	61,677	36.0 (51.7–52.6)	

*Sex*				
Female	120,319	46,112	38.3 (54.1–76.3)	*p* = 0.002
Males	85,819	30,190	35.2 (48.3–99.9)	

*Year*				
2014	20,237	7,480	37.0 (49.3–51.5)	*p* < 0.001
2015	31,799	11,339	35.7 (53.2–54.9)	
2016	41,392	14,636	35.4 (53.8–55.4)	
2017	37,970	14,284	37.6 (46.5–50.1)	
2018	38,165	14,423	37.8 (49.4–51.8)	
2019	36,575	14,140	38.7 (49.7–52.1)	
Total	206,138	76,302	37.0	

**Table 6:** Summary of patients by age groups who were HBV-infected, had natural and vaccine-induced immunity, or were at risk of HBV infection in Gauteng Province, South Africa, from 2014 to 2019 (*p*  <  0.001).

Interpretation	Age groups *n* (%)				Total
	<5 years	5–12 years	13–24 years	≥25 years	
HBV-infected: HBsAg pos; anti-HBc total pos; anti-HBs neg	21 (2.0)	42 (3.1)	272 (4.2)	3,431 (8.0)	3,766 (7.0)
HBV naturally acquired immunity: HBsAg neg; anti-HBc total pos; anti-HBs pos	113 (10.0)	45 (3.4)	541 (8.3)	11,188 (26.0)	11,887 (23.0)
HBV vaccine immunity: HBsAg neg; anti-HBc total neg; anti-HBs pos	656 (57.0)	686 (51.5)	1,757 (27.0)	4,425 (10.0)	7,524 (14.0)
HBV seronegative group: HBsAg neg; anti-HBc total neg; anti-HBs neg	368 (31.0)	560 (42.0)	3,952 (60.5)	24,524 (56.0)	29,404 (56.0)
Total	1,158 (2.0)	1,333 (2.5)	6,522 (12.4)	43,536 (83.0)	52,581 (100.0)

## Data Availability

The Microsoft Excel spreadsheet data used to support the findings of this study may be released upon application to the National Health Laboratory Services Central Data Warehouse, a division of NHLS Academic Affairs, and Research Management System, who can be contacted at CDWOffice@nhls.ac.za or collen.mukhithi@nhls.ac.za.

## Supplementary Materials

Supplementary Materials Supplementary Table 1: the annual number of patients tested for HBsAg, anti-HBc total, anti-HBc IgM, and anti-HBs by age group from 2014 to 2019. Supplementary Table 2: distribution by age groups, sex, and tests by year of patients who were categorized by HBsAg, anti-HBc total, and anti-HBs as HBV-infected, naturally-acquired, and vaccine-induced HBV immunity and HBV seronegative in Gauteng Province, South Africa, from 2014 to 2019.
