# Supplementary material for: Hepatitis B Virus Immunity Gap: A Six-Year Laboratory Data Review of Hepatitis B Serological Profiles in Gauteng Province, South Africa
Source: Adv Virol. 2023 May 17;2023:6374874. doi: 10.1155/2023/6374874 (PMC10208757; doi:10.1155/2023/6374874)
Supplement: Supplementary Materials — Supplementary Table 1: the annual number of patients tested for HBsAg, anti-HBc total, anti-HBc IgM, and anti-HBs by age group from 2014 to 2019. Supplementary Table 2: distribution by age groups, sex, and tests by year of patients who were categorized by HBsAg, anti-HBc total, and anti-HBs as HBV-infected, naturally-acquired, and vaccine-induced HBV immunity and HBV seronegative in Gauteng Province, South Africa, from 2014 to 2019. [file 6374874.f1.zip › Supplementary Table 1.docx]

**Supplementary Table 1:** The annual number of patients tested for HBsAg, anti-HBc Total, anti-HBc IgM and anti-HBs by age group, 2014 to 2019.

|  | **Yr** | **<5** | | | **5-12** | | | | **13-24** | | | | **≥25** | | | | | | **Total** | | | |  |
| --- | --- | --- | --- | --- | --- | --- | --- | --- | --- | --- | --- | --- | --- | --- | --- | --- | --- | --- | --- | --- | --- | --- | --- |
|  |  | **n** | **%** | | **n** | | **%** | | **n** | | | **%** | **n** | | | | **%** | | **N** | | **%** | |  |
| **HBsAg** | **2014** | 1,091 | 12.0 | | 1,093 | | 10.0 | | 6,071 | | | 5.0 | 41,708 | | | | 4.4 | | **49,963** | | 5.0 | |  |
|  | **2015** | 1,510 | 16.0 | | 1,830 | | 17.0 | | 16,543 | | | 13.0 | 127,407 | | | | 13.5 | | **147,290** | | 13.0 | |  |
|  | **2016** | 1,573 | 17.0 | | 2,011 | | 18.0 | | 23,705 | | | 18.0 | 182,611 | | | | 19.0 | | **209,900** | | 19.0 | |  |
|  | **2017** | 1,810 | 19.0 | | 2,124 | | 20.0 | | 25,712 | | | 20.0 | 196,798 | | | | 21.0 | | **226,444** | | 21.0 | |  |
|  | **2018** | 1,722 | 19.0 | | 1,855 | | 17.1 | | 24,250 | | | 18.0 | 178,400 | | | | 19.0 | | **206,227** | | 19.0 | |  |
|  | **2019** | 1,562 | 17.0 | | 1,951 | | 18.0 | | 35,071 | | | 27.0 | 217,153 | | | | 23.0 | | **255,737** | | 23.0 | |  |
|  | **Total** | **9,268** | **1.0** | | **10,864** | | **1.0** | | **131,352** | | | **12.0** | **944,077** | | | | **86.0** | | **1095,561** | | **100.0** | |  |
|  |  |  | |  |  | | |  | |  | |  | |  | |  | | |  |  | | |  |
| **Anti-HBc Total** | **2014** | 475 | | 22.7 | 550 | | | 26.7 | | 2,095 | | 19.1 | | 11,486 | | 14.6 | | | **14,606** | 15.6 | | |  |
|  | **2015** | 464 | | 22.2 | 520 | | | 25.2 | | 2,583 | | 23.6 | | 16,285 | | 20.7 | | | **19,852** | 21.2 | | |  |
|  | **2016** | 439 | | 21.0 | 448 | | | 21.7 | | 2,726 | | 24.9 | | 19,342 | | 24.6 | | | **22,955** | 24.5 | | |  |
|  | **2017** | 254 | | 12.1 | 230 | | | 11.1 | | 1,392 | | 12.7 | | 11,040 | | 14.0 | | | **12,916** | 13.8 | | |  |
|  | **2018** | 243 | | 11.6 | 158 | | | 7.7 | | 1,095 | | 10.0 | | 10,295 | | 13.1 | | | **11,791** | 12.6 | | |  |
|  | **2019** | 217 | | 10.4 | 157 | | | 7.6 | | 1,066 | | 9.7 | | 10,151 | | 12.9 | | | **11,591** | 12.4 | | |  |
|  | **Total** | **2,092** | | 2.0 | **2,063** | | | 2.0 | | **10,957** | | 12.0 | | **78,599** | | 84.0 | | | **93,711** | 100.0 | | |  |
|  |  |  | |  |  | |  | | |  | |  | |  | | | |  |  | |  | |  |
| **Anti-HBc IgM** | **2014** | 377 | | 12.0 | 297 | | 11.0 | | | 1,843 | | 7.3 | | 13,974 | | | | 7.0 | **16,491** | | 7.0 | |  |
|  | **2015** | 506 | | 16.0 | 500 | | 18.0 | | | 4,441 | | 18.0 | | 33,780 | | | | 16.0 | **39,227** | | 16.0 | |  |
|  | **2016** | 558 | | 18.0 | 525 | | 19.0 | | | 5,519 | | 22.0 | | 45,593 | | | | 22.0 | **52,195** | | 22.0 | |  |
|  | **2017** | 617 | | 19.0 | 593 | | 21.0 | | | 5,956 | | 24.0 | | 49,638 | | | | 24.0 | **56,804** | | 24.0 | |  |
|  | **2018** | 558 | | 18.0 | 435 | | 15.6 | | | 4,370 | | 17.0 | | 36,953 | | | | 18.0 | **42,316** | | 18.0 | |  |
|  | **2019** | 538 | | 17.0 | 438 | | 16.0 | | | 3,071 | | 12.0 | | 28,157 | | | | 13.0 | **32,204** | | 13.0 | |  |
|  | **Total** | **3,154** | | **1.3** | **2,788** | | **1.2** | | | **25,200** | | **10.5** | | **208,095** | | | | **87.0** | **239,237** | | **100.0** | |  |
|  |  |  | |  |  |  | | | |  |  | | | |  | | |  |  | | |  | |
| **Anti-HBs** | **2014** | 545 | | 12.4 | 642 | 14.5 | | | | 2,866 | 11.4 | | | | 16,184 | | | 9.4 | **20,237** | | | 10.0 | |
|  | **2015** | 701 | | 15.9 | 760 | 17.1 | | | | 4,286 | 17.0 | | | | 26,052 | | | 15.1 | **31,799** | | | 15.0 | |
|  | **2016** | 731 | | 16.6 | 873 | 19.7 | | | | 5,283 | 20.9 | | | | 34,505 | | | 20.1 | **41,392** | | | 20.1 | |
|  | **2017** | 741 | | 16.8 | 761 | 17.1 | | | | 4,659 | 18.5 | | | | 31,809 | | | 18.5 | **37,970** | | | 18.4 | |
|  | **2018** | 885 | | 20.1 | 688 | 15.5 | | | | 4,193 | 16.6 | | | | 32,399 | | | 18.8 | **38,165** | | | 18.5 | |
|  | **2019** | 803 | | 18.2 | 718 | 16.2 | | | | 3,942 | 15.6 | | | | 31,112 | | | 18.1 | **36,575** | | | 18.0 | |
|  | **Total** | **4,406** | | 2.1 | **4,442** | 2.2 | | | | **25,229** | 12.2 | | | | **172,061** | | | 83.5 | **206,138** | | | 100.0 | |
