# Supplementary material for: Hepatitis B Virus Immunity Gap: A Six-Year Laboratory Data Review of Hepatitis B Serological Profiles in Gauteng Province, South Africa
Source: Adv Virol. 2023 May 17;2023:6374874. doi: 10.1155/2023/6374874 (PMC10208757; doi:10.1155/2023/6374874)
Supplement: Supplementary Materials — Supplementary Table 1: the annual number of patients tested for HBsAg, anti-HBc total, anti-HBc IgM, and anti-HBs by age group from 2014 to 2019. Supplementary Table 2: distribution by age groups, sex, and tests by year of patients who were categorized by HBsAg, anti-HBc total, and anti-HBs as HBV-infected, naturally-acquired, and vaccine-induced HBV immunity and HBV seronegative in Gauteng Province, South Africa, from 2014 to 2019. [file 6374874.f1.zip › Supplementary Table 2.docx]

**Supplementary Table 2:** Distribution by age groups, sex and tests by year of patients who were categorized by HBsAg, anti-HBc Total and anti-HBs as HBV infected, naturally-acquired and vaccine-induced HBV immunity and HBV seronegative in Gauteng Province, South Africa, from 2014 to 2019.

| **Variable** | **N** | **HBV Infected** | | | **Naturally-acquired Immunity** | | | **Vaccine-induced Immunity** | | | **HBV seronegative** | | |
| --- | --- | --- | --- | --- | --- | --- | --- | --- | --- | --- | --- | --- | --- |
|  |  | **n** | **% (95% CI)** | ***p* value** | **n** | **% (95% CI)** | ***p* value** | **n** | **% (95% CI)** | ***p* value** | **n** | **% (95% CI)** | ***p* value** |
| **Age group** |  |  | | |  | | |  | | |  | | |
| 0-4 | 1,222 | 21 | 1.7 (1.1-2.6) | <0.001 | 113 | 9.3 (7.8-11) | <0.001 | 656 | 53.9 (20.9-56.5) | <0.001 | 368 | 30.1 (27.6-32.8) | <0.001 |
| 5-12 | 1,364 | 42 | 3.1 (2.3-4.1) |  | 45 | 3.3 (2.5-4.4) |  | 686 | 50.3 (47.6-52.9) |  | 560 | 41.1 (38.5-43.7) |  |
| 13-24 | 6,724 | 272 | 4.1 (3.6-4.5) |  | 541 | 8.1 (7.4-8.7) |  | 1,757 | 26.1 (25.1-27.2) |  | 3,952 | 58.8 (57.6-60.0) |  |
| **Age group** |  |  | | |  |  |  |  | | |  | | |
| 0-24 | 9,310 | 335 | 3.6 (3.2-4.0) | <0.001 | 699 | 7.5 (7.0-8.1) | <0.001 | 3,099 | 33.3 (32.3-34.3) | <0.001 | 4,880 | 52.4 (51.4-53.4) | 0.697 |
| >24 | 46,984 | 3,431 | 7.3 (7.1-7.5) |  | 11,188 | 23.8 (23.4-24.2) |  | 4,425 | 9.4 (9.2-9.7) |  | 24,524 | 52.2 (51.7-52.7) |  |
| **Gender** |  |  | | |  | | |  | | |  | | |
| Male | 23,995 | 2,280 | 9.5 (9.1-9.9) | <0.001 | 5,435 | 22.7 (22.1-23.2) | <0.001 | 2,704 | 11.3 (10.9-11.7) | <0.001 | 11,677 | 48.7 (48.0-49.3) | <0.001 |
| Female | 32,299 | 1,486 | 4.6 (4.4-4.8) |  | 6,452 | 20.0 (19.5-20.4) |  | 4,820 | 14.9 (14.5-15.3) |  | 17,727 | 54.9 (54.3-55.4) |  |
| **Year** |  |  | | |  | | |  | | |  | | |
| 2014 | 8,216 | 481 | 5.9 (5.4-6.4) | <0.001 | 1,613 | 19.6 (18.8-20.5) | <0.001 | 1,548 | 18.8 (18.0-19.7) | <0.001 | 4,143 | 50.4 (49.3-51.5) | <0.001 |
| 2015 | 13,431 | 874 | 6.5 (6.1-6.9) |  | 2,539 | 18.9 (18.3-19.6) |  | 1,932 | 14.4 (13.8-15.0) |  | 7,269 | 54.1 (53.3-55.0) |  |
| 2016 | 15,242 | 1,062 | 7.0 (6.6-7.4) |  | 3,063 | 20.1 (19.5-20.7) |  | 1,718 | 11.3 (10.8-11.8) |  | 8,321 | 54.6 (53.8-55.4) |  |
| 2017 | 6,178 | 525 | 8.5 (7.8-9.2) |  | 1,412 | 22.9 (21.8-23.9) |  | 743 | 12.0 (11.2-12.9) |  | 2,951 | 47.8 (46.5-49.0) |  |
| 2018 | 6,491 | 395 | 6.1 (5.5-6.7) |  | 1,607 | 24.8 (23.7-25.8) |  | 747 | 11.5 (10.8-12.3) |  | 3,286 | 50.6 (49.4-51.8) |  |
| 2019 | 6,736 | 429 | 6.4 (5.8-7.0) |  | 1,653 | 24.5 (23.5-25.6) |  | 836 | 12.4 (11.6-13.2) |  | 3,434 | 51.0 (49.8-52.2) |  |
| **Total** | **56,294** | **3,766** | **7.0** |  | **11,887** | **21.1** |  | **7,524** | **13.4** |  | **29,404** | **52.2** |  |
